# Exploring hospital certification processes from the certification body’s perspective - a qualitative study

**DOI:** 10.1186/s12913-020-05093-w

**Published:** 2020-03-23

**Authors:** Dag Tomas Sagen Johannesen, Siri Wiig

**Affiliations:** 1 Department of Economic, risk management and planning, University of Stavanger, 4036 Stavanger, Norway; 2 Department of Health and Nursing Science, University of Agder, 4604 Kristiansand, Norway; 3 SHARE-Center for Resilience in Healthcare, Department of Quality and Health Technology, Faculty of Health Sciences, University of Stavanger, 4036 Stavanger, Norway

**Keywords:** Certification, ISO 9001, Auditor, Surveyor, External assessment

## Abstract

**Background:**

Hospital certification is an external assessment mechanism to assure quality and safety systems. Auditors representing the certification body play a key role in certification processes, as they perform the assessment activities and interact with the involved healthcare organizations. There is limited knowledge about the approaches and methods that auditors use, such as role repertoire, conduct, and assessment practice. The purpose of this study was to explore auditors’ practice in hospital certification processes, guided by the following research questions: What styles do auditors apply in hospital certification processes, and how do auditors perceive their role in hospital certification processes?

**Methods:**

The study was performed in two stages. In the first stage, non-participant observations (59 h) were conducted, to explore the professional practice of three lead auditors in certification processes of Norwegian hospitals. In the second stage, semi-structured interviews were conducted with these three observed lead auditors. The role repertoires and conducts identified were analyzed by using a deductive approach according to a surveyor (equivalent with auditor) styles typology framework.

**Results:**

Two distinct auditor styles (“explorer” and “discusser”) were identified among the three studied auditors. Both styles were characterized by their preference for an opportunistic and less structured type of interview practice during certification audits. All three auditors embedded a guiding approach (reflections about findings, stimulate improvements, experience transfer from other industries) to their perception and practice of certification audits, interacting with the auditees. The use of group interviews instead of individual interviews during certification audits, was the rule of their professional practice.

**Conclusion:**

The auditors’ perceptions and styles demonstrated a multifaceted certification reality, in contrast to what is often presumed as consistent, stringent and independent practices. These findings may have implications for reliability judgements when developing hospital certification programs, and for the refinement of the current framework used here to study the different auditing practices.

## Background

ISO (International Organization for Standardization) 9001 Certification is used for external assessment of quality and safety systems in health care. It is often associated with accreditation and other external assessment programs for health care organizations [1–6]. In these programs, external assessment bodies collect evidence to assess whether organizational systems and performances are following recognized standards and most often provide a certificate on successful compliance with the standard. In addition, many programs intend to add value and support improvement in organizations. Auditors, equivalent with surveyors, inspectors, assessors, evaluators, or visitors in other programs [6–8], are key to certification, as they perform the assessments and interact with the involved healthcare organizations. In ISO 9001 certification, auditors assess conformity to requirements for quality management systems, and as with most other external assessment strategies their main methods to collect information include interaction with the certified organization through interviews, observations, and review of documentation and records [9, 10].

Concerns have been raised related to arguments of scarce evidence of the benefits from external assessment programs, and the wide use and amount of resources that healthcare services allocate to these programs [11–14]. There are contrasting views about accreditation programs among health care professionals. Some view these programs as effective for development of organizational processes and patient safety, while others have concerns about the bureaucratic burden, the financial and human resources that are required, and the efforts to meet a large number of standards [15]. There is scarce evidence of the impact of care outcome measures [13, 16]. However, previous research has documented how accreditation has potential impact on organizational processes, changes in professional practice, and cultural change concerned with quality of care [17–19] and that certification and accreditation may be better than no external assessment when associated to hospital outputs and quality and safety structures [3, 20]. Furthermore, previous research has demonstrated that hospital accreditation and ISO 9001 certification were not significantly associated with evidence-based clinical care, but had significant benefits to patient safety systems and processes, such as clinical leadership and clinical review [21]. Other studies showed how admission to a fully accredited hospitals can be associated with a lower 30-day mortality risk compared to admission at partially accredited hospitals [22] and that a frequent accreditation cycle may have positive impact on maintaining hospital quality [23].

Despite growing evidence of the effects from certification and accreditation in healthcare, little is known about practices and mechanisms involved in the interactional processes between assessment bodies and healthcare organizations in external assessments. In external assessments systems, such as ISO certification, the auditors’ experience, selection of auditors, training, support and motivation may influence the performance, style, and reliability of the auditing (assessment) practices [6, 8, 24–26]. The approach and methods that auditors use in their assessments and verification processes, such as role repertoire, auditor’s conduct (e.g., inspection or guidance) and assessment practice need further exploration [15, 25, 27–29]. Exploring these matters may identify elements that can be beneficial for training, development and consistency of certification programs both within and between certification bodies, and for further research. For healthcare organizations and policy makers it may benefit from the transparency and insight into these widely used means, often involving for-profit certification bodies, to monitor, assure and improve performance in healthcare. Transparency and insight make it possible to better judge for what grounds certification decisions are made, and for fairness, and reliability. Insight into auditing practices is also important in order to meet requests for more flexible and context dependent external evaluation programs that are relevant for future changes in healthcare and to meet demands for user involvement [30].

The purpose of this study was to explore the audit practice as perceived and performed by auditors in one certification body involved in hospital certification processes in Norway. The following two research questions guided this study: (i) What styles do auditors apply in hospital certification processes, and (ii) how do auditors perceive their role in hospital certification processes?

By exploring the ways in which auditors perceive and perform their role in three hospital certification processes this study contributes with valuable insights into the practices and approaches of a for-profit certification body that often can be challenging to get access to. The exploration reveals different role repertoires and professional conduct among the auditors from the certification body. We discuss how this influences further research and development of future certification processes, and highlight the implications for policy makers.

### ISO 9001 certification: the normative framework and the certification process

ISO 9001 quality management system certification is a third-party conformity assessment (audit) against requirements in the international standard ISO 9001 *Quality Management Standard – Requirements* [31, 32]. In health care, ISO 9001 certification is applied to entire organizations and to their individual departments. The requirements for certification bodies and their auditors are stipulated in the international standard ISO/IEC 17021 *Conformity assessment -- Requirements for bodies providing audits and certification of management systems* [9, 33, 34]. The standard intends to ensure that certification bodies operate management system certification in a competent, consistent and impartial manner, and “the overall aim of certification is to give confidence to all parties that a management system fulfills specified requirements” [34]. The standard emphasizes consistency both in audit program processes and in the reporting of results, and certification decisions should be based on objective evidence. Impartiality is described as a necessity for certifications that instills confidence [34, 35], and the management and control of impartiality are required for certification bodies. The normative standard for third-party conformity assessments is underpinned by the guiding principles in the generic guidelines for auditing management systems [36].

An audit program has a three-year audit cycle: initial certification, surveillance audits in the first and second years, and re-certification in the third. The on-site audit activities include an opening meeting to introduce the audit team, confirm the audit plan and scope, and to verify the procedures and communication that are used during the audit. The next phase is to collect and verify information pertinent to the audit objectives, scope and criteria and prepare audit conclusions. Methods of collecting information include interviews, observation of processes and activities, and review of documentation and records. The ISO standards presents interviews as one of the main methods to collect information, but do not describe the method in detail or explicitly refer to one respondent interviews or suggest group interviews [9, 10, 34, 36]; nor do the guidelines that recommend good practices for all elements of conformity assessment [37]. Finally, the audit team holds a closing meeting to present and discuss conclusions and non-conformities and agree on follow-up actions.

### Auditor typology framework

**Fig. 1:**
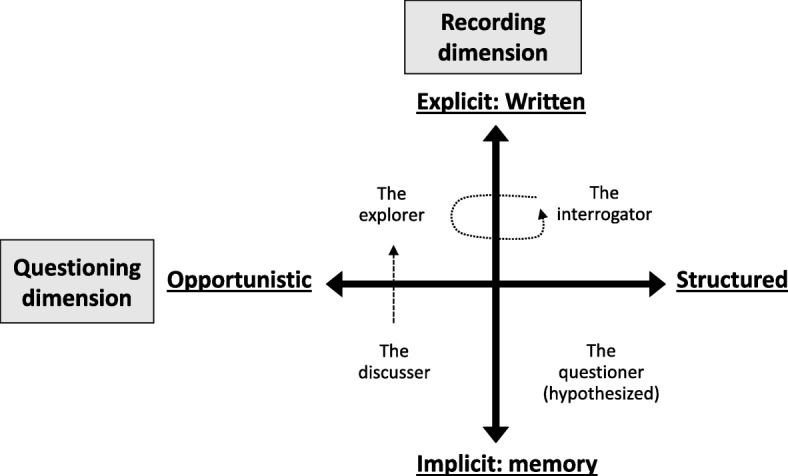
Auditor style typology adopted from Greenfield, Braithwaite & Pawsey [24] with permission. The arrows show that the interrogator sporadically used opportunistic questioning, and that the discusser took unstructured notes after the interviews

Several typologies explain regulatory institutional practice in the regulator-regulatee encounter, like characteristics of the regulated organizations [38]; regulator’s perception [39]; inspector’s inconsistency [40]; and types of relational signals [41]. In this study, we apply a typology framework developed from research on auditors in health care accreditation in Australia [24] to explore and analyze the styles that auditors in ISO certification apply. The framework was developed from observations on how a team of three auditors undertook their auditing practice in a small health organization providing general and specialist medical and surgical services [24]. The researchers’ observations focused on assessing against the standard, education for improvement, and learning for transfer of knowledge to others. Based on the observed differences in the auditors’ interview practice, three distinct styles were identified: *interrogator*, *explorer* and *discusser*, and one hypothesized style: *questioner*. These styles were categorized within two dimensions (see Fig. 1): questioning (structured vs. opportunistic), and recording (explicit: written vs. implicit: memory).

#### The interrogator

The auditor conducts interviews in a formal and structured question-and-answer manner, and the answers are systematically recorded as she/he proceeds. Questions may be prepared in advance based on the standard. The interrogator had sporadically short periods of opportunistic questioning as approached by *explorer*.

#### The explorer

The explorer conducts a more opportunistic interview. The explorer begins with open-ended questions, and takes unstructured notes. This auditor typically comments about what she/he has learnt and can use elsewhere. The explorer is less inclined to engage in the educational component of audits.

#### The discusser

The discusser prefers a more interactive interview that is like a discussion. All the three elements -- assessment, education, and learning -- are implicitly a part of the discussion. The discusser took unstructured notes, like the *explorer*, after the interviews.

#### The hypothesized questioner

The questioner conducts a structured interview. The recordings are conducted implicitly, making this kind of interview seem less formal than the interrogator’s.

## Methods

### Design

The study was a qualitative explorative single case study [42] of a certification body interacting in certification processes in hospitals in Norway. The case study had three embedded units of analysis consisting of three lead auditors from the same certification body that performed certification audits in three different hospital organizations. We explored how the auditors conducted and perceived their certification processes.

### Data sources and collection

Data collection was performed in two stages (see Table 1) from autumn 2012 until spring 2013. First, we explored the different role repertoires and professional conduct among three lead auditors from the same certification body by conducting non-participant observations in third-party conformity assessments (certification audit) and then semi-structured interviews were conducted with the same lead auditors. The three auditors were purposefully selected from one certification body in Norway. The certification body was one of four certification bodies in Norway that were approved by accreditation^1^ to perform external third-party conformity assessments and certifications of hospitals according to the ISO 9001:2008 standard.

**Table 1:** Data Collection and Sources

	Data collection	Auditors	Location/organization	Duration
**1. stage**	Non-participant observations of three separate conformity assessments (ISO 9001:2008 certifications)	Auditor 1: lead auditor (in training)	Clinic for internal service	2 days (about 15 h.)
		Auditor 2: lead auditor (5–10 years auditor experience)	Full hospital	3 days (about 22 h.)
		Auditor 3: lead auditor (independent subcontractor, > 20 years auditor experience)	Emergency department	3 days (about 22 h.)
**2. stage**	Semi-structured interviews	Auditors 1, 2 and 3	Auditors 1 and 3: The central office of the certification body Auditor 2: By telephone	45–75 min per interview

Stage 1: The first author conducted about 59 h of non-participant observation in three separate certification processes according to the *ISO 9001:2008 Quality management systems – requirements* revision of the standard. The first author followed three audit teams, all from the same certification body. The certification body received a written and oral invitation to participate in the study prior to observations and interviews. The certification body further identified the three auditors and was the first to ask them to participate in the study. The first author followed the lead auditors through all on-site audits, and usually sat at the table with those involved in the audit during the audit interviews. All notes were taken openly, and the observations focused on the conduct of the lead auditors (team leaders) during their interviews and conversations (review process) with representatives from the organization under certification. The two dimensions in the auditor style typology -- questioning and recording -- guided the observations and note taking. The observations did not focus on the auditors’ on-site walk-arounds, where they had informal talks with different hospital staff and reviewed physical processes and activities in the hospital.

Stage 2: Some weeks later, semi-structured interviews [43] were conducted with all three lead auditors who had been observed. The first author conducted the interviews. Each interview lasted from 45 to 75 min. The interview guide centered around three main issues: First, the interview subjects’ role and the certification body’s approach to ISO 9001 certification; second, the certification processes; and third, ISO 9001 certification and its relationship to the health care regulation. Open questions were generally used, followed by either preplanned or ad hoc probing questions, in order to help interview subjects to recall and tell more detailed stories. All interviews were tape recorded and transcribed verbatim.

### Setting and participants for observations

The first and second observations took place in organizations that were changing certification body and were therefore undergoing a full review in order to become re-certified. Hence, it was the first time for the lead auditors to survey these organizations. The first survey was performed in a clinic for internal service. The second survey took place in a hospital where the objective was to certify the whole hospital. Many of the departments within this hospital were already ISO 9001 certified by another certification body, and were included in the overall certification process. The survey was performed as an on-site document review, whose objective was to review the management system documents and records, and their adequacy in terms of the requirements in the ISO standard. This assessment differed from the others in our study as it did not include an on-site observation of activities and the work environment. On-site observation was planned at a later stage. The third survey was conducted in an emergency department with a three-year certification history, and was now part of a re-certification process.

Personnel from the organizations present during the interviews were managers, management representatives, and staff responsible for following up the organizations’ management systems. Technical expert auditors were present for the assessments of specialized departments, such as those responsible for technical equipment, cleaning or medical specialties.

### Analysis

All of the data material from the observational field notes and the transcribed interviews was subjected to theoretical (deductive) thematic analysis [44, 45] of each of the three auditors. The two dimensions in the auditor style typology [24] – questioning ((a) structured vs. (b) opportunistic), and recording ((c) explicit: written vs. (d) implicit: memory)– were employed as predefined themes to categories data from the observations, in order to identify the auditors’ conduct that matched or contradicted with the auditor styles in the framework. Further, data from the observations were explored according to the predefined themes related to the opposites between (e) assessing conformity against requirements, focusing on retrospective auditing practices (inspect, control), and/or (f) quality improvement work, focusing on prospective auditing practices (guidance, educate, transfer experiences, give advice). The same themes were used to analyze data from interviews in order to reveal the auditors perception of their auditing approach. NVivo 10 was used to explore and thematically analyze the interview data. Data from the interviews were then related to data from the observations, and compared in a reflexive manner to explore whether the auditing style and conduct as observed were concurrent with the auditors perceived approach. The analysis was conducted for each auditor, in order to highlight the identified auditing styles and the themes emphasized in perceptions and practices, before we compared the themes in the analytical framework across auditors to illustrate commonalities and differences.

To ensure trustworthiness in the analysis we conducted member checks by sending a draft of the paper to all three auditors. The auditors were able to comment if they recognized the description of themselves, the use of data and clarify misinterpretations of facts and figures. One auditor gave her response by phone, another responded both by phone and e-mail, and the last by e-mail. Only minor changes to the draft was made after the member check.

## Results

In the following section, findings from the three auditors are presented. For each auditor, we present his or her perception of how he or she approached the audit interview situation; then we present the observation findings from the audit interview; further, we present what the auditors perceived as their main tasks according to the dimensions of assessing conformity or stimulating improvement; and finally, summarize and compare the auditors’ style and perceived auditing approach.

### The explorer: auditor 1

The auditor cited dialogue in her auditor-auditee encounter as an important means of collecting information and for stimulating and guiding improvement. As part of that audit dialogue, she asked deeper questions to collect additional information:

It’s part of that dialogue. First you check out: “How do you do it?” Get a clear view of that. Then you ask if they actually experience that their approach is appropriate. What actually do work, the way they do it, and what do not. And based on that you will say: I have seen others who have a slightly different approach, so perhaps we can adjust a little bit. […] [T] hey follow the book since they fulfill the minimum requirements, but should they, when they have those [the requirements] in place, consider whether they can get more out of that arena. (Auditor 1).

During the on-site assessment in the clinic for internal services, the auditor performed all her interviews with groups of staff from the different departments. The groups never had fewer than four people. The respondents were usually a manager or middle manager, the hospital’s contact person/quality manager, and one or two others “relevant personnel” (the term used in the agenda) from the departments under assessment. All interviews were held in the same room. The auditor used a template to highlight the main requirements in the ISO 9001 standard against which she assessed conformity, and to structure the progress during the interview. The auditor recorded what was said, either in her notebook or on the template sheet. A projector was used in most of the interviews to review and discuss documents and recordings, like policies, objectives, plans and procedures. The auditor usually started with an open-ended question, such as “Can you tell me about the management system?” She invited a conversation about the topic, or had the respondents explain documents on the screen. If the respondents mentioned something that caught her interest, she either asked for verification or a probing question. An example was observed when the auditor asked the auditee about the control of errors and non-conformities. A vague answer from the respondents led her to ask for more explicit documentation, and to see examples of how they correct errors and non-conformities. She directed most of her questions to all the respondents in the room, and was usually answered by the manager (often a middle manager) who was responsible for that topic. The others (often one or two employees from the department and the hospital’s contact person/quality manager) followed up if the manager seemed to have left something out of the answer.

According to Auditor 1’s perception her main task was to bring added value to the organization. This was not just related to the general understanding of the expected added value from third-party certification (e.g., increased legitimacy), but also to organizational development as part of the certification process. Again, the auditor opened a dialogue.

When we are out, they want us to create added value for the customer, and help the customer to become better at managing risks in relation to their core areas. […] Moreover, in my decision-making process I will then review and ask, in a dialogue with the customer: “What do you see as challenging?”; “Where do you think the problems lie?”; That’s just as important …, that dialogue is very important. (Auditor 1).

The perception of improvement was emphasized when auditor 1 described her approach within the inspection-guidance axis. She perceived concern over the balance of not adopting a consultancy approach.

You largely control, but at the same time you’re also a guide. I do not think it is possible to see them isolated. So, you control in the sense that you can kind of check out if things are in place - yes or no. However, since improvement is as central as it is, then together with the customer, in that dialogue, it is to identify things where one in advantage might take small steps to raise oneself further ahead. That becomes the guidance role. I think they go like hand in glove. At the same time, it is important in that guidance role, that you do not give very clear recipes on how things should be done. (Auditor 1).

### The explorer: auditor 2

The next auditor stressed the importance of asking questions to elicit information that was needed for the audit. She also perceived her manner of asking questions as an explorative encounter, where the intention was to foster self-reflection.

The most important tool is to ask question, and then to be shown what you have requested. Maybe they do have it, maybe they don’t. And then use what comes to the table in the best possible manner. We may not always follow the preplanned agenda, because one sees that there are strengths in some areas, or weaknesses in other areas where you need to go in-depth. Because, that’s what gives results, getting to the core, and perhaps especially the way one asks questions, so that they themselves see it. It should be themselves who sees it- those are the best audits. When they get a wake-up-call that make them see that this is useful, and they see where they can get better. (Auditor 2).

In her interview strategy, auditor 2 perceived it as important that the respondents were informed, knew the purpose of the session, and felt safe during the audit interview:

Moreover, it is often appropriate that they are not alone, but that there are two, three of them, so they can ask each other. […] If there are several findings of nonconformity they can feel unfortunate, and think that if someone else had answered maybe it would have been different. They try to represent the organization and they want to be good. That’s what we all want to. (Auditor 2).

During her three-day document review of the hospital’s quality management system, the auditor always performed the interview with at least two people from each department, often a manager and a staff member who was involved in quality development in the department. Observations showed that she used a template to guide the interview, but unlike the first auditor she sporadically recorded what she heard and observed. She used a projector during the interviews to review and discuss documents and recordings, like policies, objectives, plans and procedures. Like the first auditor she also often started with an open question when shifting to a new topic. A significant difference from the first auditor was that the second auditor often raised questions about solutions and self-reflection, especially when she observed potential improvements or non-conformities, like “What is a challenge here?” “How and where would a nurse look for this procedure?” If respondents asked for feedback or suggestions about their management systems, the response was often “What is useful for you?” These answers encouraged reflections instead of giving direct suggestions or advice, and also indicated the importance of quality systems as useful and not for the systems in itself. When interviewing the top management, the auditor seemed to base her arguments upon the logic of the quality management standard, as when she was interviewing the top management, represented by the managing director, the vice managing director and the management representative. The observations showed that vice managing director entered in a hurry and asked if it was necessary for him to participate. He agreed to “take part in the beginning” after getting a very short explanation of the objectives for the meeting. The auditor went through the organization’s quality policy, quality objectives, management review and the like in a dialogue-based approach with the respondents, often referring to, and explaining requirements in the ISO standard. The vice managing director turned out to be the most engaged in the whole meeting, and stated that the ISO 9001 management system approach visualized challenges that clearly had improvement potential.

The auditor perceived verification to be her most important task, which entailed to assess if there was consistency between the systems organizations have and the practices they perform, and to look for improvement and what works well. Her focus on improvement and the certification process was also perceived as valuable from her perspective:

Then it’s about getting a review of the system. What we are talking about …; I can see that awareness, learning, and systematic improvement is helpful for them, because they use their own material to understand these aspects within a context. This is what I may experience is of greatest value, because the report [they receive from us] is short. The process that it entails is perhaps just as important for those involved, so that they over time constantly strive to understand more of the system, systematics, improvement opportunities, in their own organization. (Auditor 2).

Observations and interviews showed that improvement was a central theme of her audit practice. It was embedded in a focus upon motivational factors to bring about organizational improvement. She expressed respect for the limited possibility of her getting a total picture of the organization during a couple of days, and kept in mind that she was not there to tell the organization how things should be done or give advices, but to a great extent provide support for good practice and identify opportunities by giving examples from best practices elsewhere. She perceived that the control dimension was about 30% of her auditing practice. Auditor 2 stated that there might be some differences in the way she performed audits in hospitals than in other sectors, and suggested that underdeveloped management systems might have been a reason for her motivational approach rather than verification.

### The discusser: auditor 3

When auditor 3 conducted her interviews, she wanted the participants to be engaged and to contribute to the discussions. Both revision and sharing of experiences were embedded in her dialogue strategy. She strategically used her interview to communicate requirements and meet key personnel in daily practice. As she expressed:

[T] he way I’ve started now, because of what I have experienced in the health sector, where they have placed very much on the quality managers. So, when I put management processes on the [audit] agenda, […] I then ask if all the persons who participates in the management review [from the organization] can participate. Then, it is not just the head of department, or director of administration, or... So, when we assess the minutes from meetings and things like that, all participants should have a possibility to participate – and for me to be able to ask them about their expectations of the system and to get to know them, and so on. So, there’s a direct auditor role. But they have to think themselves to speak. They have to make their, their contributions then -- more active. (Auditor 3).

Auditor 3 started her three-day recertification of an emergency department by claiming in the opening meeting that she preferred an audit that was based on open dialogue. She often repeated this at the start of every interview. She emphasized that she wanted no surprises in the closing meeting, and that everything should be clarified during the assessment. The audit was arranged so that all the interviews were held in locations where the personnel did their daily work. She said that she wanted to talk to people while they were closest to where they worked. In one audit interview, observations showed that the auditor spoke with only one respondent at some times, but in all others, there were at least two or more other people present. She usually began the interview with a short introduction about the intention for the assessment. She then asked for a description of how things were done in relation to the matter to be discussed. She encouraged the staff to talk about their system, and welcomed dialogue or conversation. One respondent thought she was supposed to be asked questions, and said she had not prepared for an open or “formal” presentation about how “we do things around here.” Auditor 3 often asked direct questions about an issue that had arisen during the conversation, and was then clearly steering the interview. She alternated direct questioning with dialogue. Her point of departure was based on documents or procedures that she had read beforehand in relation to the standard, but never used the standard openly during the interview. Sometimes she drew analogies to her professional background in the process industry, and discussed good practices that she had observed elsewhere. She did not record her interviews, but occasionally took notes when there seemed to be findings related to conformities or nonconformities.

Auditor 3 perceived that the certification body expected her to add value when she performed her assessment practice. The practice of adding value seemed to be related to a guidance role. At first, auditor 3 seemed a bit reluctant to focus upon a guiding role or to give advice:

[…] because I’ve always kept that one should be objective and not give advice and so forth, but it’s a balancing act when you see that the customers are not completely familiar with the extent of the requirements in the standard. (Auditor 3).

When responding to the inspection-guidance axis she expressed an organizational reality that asked for reflexivity towards the maturity of the organization’s competence on what the ISO 9001 standard actually mean for their own management system. She stated that it is not enough “to sit in school and learn the standard” (Auditor 3), the organizations have to translate the content to their own management system as well. Auditor 3 cited examples of how the audits and certification processes drove development and change in hospitals, even though her formal role during certification was assessing the organization. For example, there were often many other control mechanisms that hospitals needed to conform to, and therefore the regular certification activities could become an arena for organizations to seek advice. The auditor perceived that sharing experiences from elsewhere was the best way to avoid giving advices and to balance the audit practice between inspection and guidance.

### Summary of themes in the findings

Overall, our findings showed that all three auditors adopted an opportunistic questioning approach, but auditor 3 was more likely to turn to a more direct and closed type of questioning than the others. The first two auditors used a template to guide their interview related to the normative standard, but none used structured pre-planned questions. There were significant differences in how the auditors recorded the interview results. The first two auditors took detailed notes during the interview - the first more frequently than the second. This showed that they were oriented towards the written side of the recording dimension. The third auditor only jotted down a few words occasionally. She was more oriented to the opposite side of the recording dimension: implicit note taking. The two first auditors adopted the conduct of the explorer, while the third adopted the conduct of the discusser. All the auditors perceived both assessment of conformity to the ISO 9001 standard and guidance for improvement as embedded parts of certification audits. They were all concerned about not giving the certified organizations specific advises on how to improve but were familiar with transfer of experiences from elsewhere and giving general guidances. Auditor 2 and 3 were also more concerned with encouraging the certified organizations own reflections of their quality management system, in order to nurture improvement. In the next section, we will discuss this in further detail.

## Discussion

In this paper, we explored auditors’ role in hospital certification. By ways of non-participant observation of audit interviews and qualitative interviews with the lead auditors, we have explored different auditor styles and how the auditors perceive their role in hospital certification processes in Norway. In the following we will discuss the findings according to the auditor typology framework [24].

### Auditors role repertoires and professional conduct – mediating opportunities for assessment and improvement

According to the normative standards, certification relies upon objectivity and consistency among auditors [27, 46]. Consistency is also a key to the reliability of certification and accreditation programs [25]. However, the normative guidelines [37] state that ISO 9001 must not be considered as a “tick-off” scheme, because the detailed requirements are only means to ensure the most important focus, which is the customers’ (or patients’) requirements. The normative frame of reference (ISO standards) takes into account that auditors get only a snapshot of what is going on in the organizations. Hospital organizations and healthcare services are complex, and the auditors should be able to comprehend that complexity in these systems during the assessments. Auditors need information to “diagnose” the management system [47] and our study demonstrated how the auditors used the standard to help gather the information that they need. Our auditors, however, took different approaches in their search for this information. All used open questions, but auditors 1 and 2 relied on structuring interviews according to the standard, while auditor 3 emphasized more an open dialog approach. Auditor 2 and 3 were more concerned with encouraging reflection and hence a higher degree of learning within the organization during the audit interview.

In a study of stakeholders’ views and experiences on accreditation survey reliability [25] the authors found that the reliability of accreditation is embedded in its technology and enacted in audit practice. Technologies like the accreditation programs themselves, workforce management and documentation have a greater influence on reliability than the conduct of auditors and the dynamics in the auditor-organization encounter. Technological factors can narrow the potential expectations and conduct and therefore also assume more consistencies, but only to a certain degree. A consistent regime within which certification auditors act, helps auditors to meet a level of reliability in complex and shifting contexts such as hospitals, but the dynamic variations are determined by the auditors’ different self-governing systems. In our study, we found that the auditors conducted their role according to the standards and expectations of their role. However, they also sometimes used their competence and experience to add value to the hospital under certification. Our results are in line with the literature on certification and accreditation programs showing an increased focus on holistic processes [12, 48] in terms of organizational development, where auditors are more involved in the improvement activities, sharing experiences, educating and giving advice. It is not just the verification itself, but a mutual process and understanding between the certification body and the hospital. These institutional changes can shape the expectations and legitimacy concerns in auditor-auditee relationships.

The auditors in this study expressed concern about giving advice, since organizational follow-up on such advices may imply that auditors will audit their own solutions (advices) at a later stage. The normative standards describe consultancy work, like advice and education, as threats to confidence in the process. The three auditors seem familiar (numbers 1 and 2 even more so than 3) with giving suggestions (not “advices” in their opinion) for improvement and transfer experiences from other hospitals, even though it may blur the strict consistency tied to the certification norms. Our results are similar to what is found in studies of regulatory models that seem to be deterrence-oriented in the first place, but more compliance-oriented at the sharp end (auditors, regulatory staff) [41, 49]. This seems to be because of the realities of “street-level” interaction where services improvement can be reached by some degree of advice or experience transfer from other sectors that are more advanced in their organizing of quality. This knowledge is important for policy makers and certification bodies, as their adaptive capacity in these roles play a key function in translating hospital certification into sound learning processes. This appears equally important to the auditors, as the certificate itself.

### Auditor styles within the surveyor style typology

Our findings show that the auditors approached and perceived their role both as assessors of compliance, while educating and stimulating improvement. The preplanned audit agenda was guided by topics from the ISO 9001 standard and directions during interviews was embedded in the requirements among all the auditors. These findings show that the structured certification program contributes to consistency, despite the differences in surveying style, and are in line with findings in other studies [24, 25].

A central proposition in the surveyor style typology is the assumption that auditors perform the same role in different ways. Gaining knowledge about the auditors’ perception of their audit role is therefore important to understand why auditors perform their role differently, especially if we are to follow the suggestions of Greenfield, Braithwaite, and Pawsey [24] about using the typology framework as a tool for training, development and assessment of auditors. Our study has explored some factors that might assist in understanding why auditors perform the same role in different ways. Greenfield, Braithwaite, and Pawsey [24] also suggest using the typology in allocation processes to ensure survey teams with either similar or mixed styles, targeting different organizational contexts. This allocation approach presumes knowledge about the effects of different audit styles upon the organization. There is no consensus on the effects of different styles in auditor-auditee encounter. Similar questions about styles have been at the heart of theoretical development in regulation theory [49–51]. According to the conformity assessment standard [34] the auditors may identify and record opportunities for improvement but refrain from suggesting the cause of nonconformities or their solutions. The auditors in this study claimed that hospitals rarely have the same comprehensive management systems that other sectors do. They perceived themselves as balancing verification with inspection, and between guidance and advice. It is difficult to see if there is a clear boundary between these two extremes, but what might seem apparent is that none of the auditors perceive or approach their role in a “highly inspectoral” manner.

Systems audits as performed in ISO 9001 certification are similar to the methods used by inspectors in the Norwegian Board of Health Supervision, who performs system audits founded in the ISO standard for audit practice [36]. The main difference is the possibilities for enforcement and the power balance. Certification bodies do not have the same possibility of escalating enforcement strategies that regulatory authorities can, and therefore have a different pressure for taking part in voluntary system improvement than what inspectors have. These aspects are often linked to independence challenges. Organizational independence is most often described as the challenge in third-party audits, but operational independence might influence the street-level practice of auditors [47]. As noted earlier, auditors depend on information. A solution to the information asymmetry between the auditors and the auditee is a more interactive and collaborative style, as adopted by the auditors in our study. Another aspect in operational independence is the epistemic dimension [47]. It is expected that clear standards imply more inspection-oriented role performance than assessment against generic standards, which is the case in ISO 9001 certification where the standard is more generic.

### Implications for the surveyor style typology and suggestions for further framework development

The auditor practice showed a considerable use of group interviews. Two auditors explained it as a strategic choice, because of respondents’ uncertainty during the assessment, an opportunity to target people who are involved in daily practice, and possibilities for reflections and awareness through discussions. Even though it seems a well-known practice, we have not been able to find suggestions about group interviews in the audit standards [33, 34, 36] that underpin the auditors’ review practice. Even individual interviews are hardly mentioned, in comparison with the amount of literature on research and evaluation methodology, where interviews are subject to rigorous scrutiny about how they are able to give or produce evidence [52–55]. We propose the use of group interviews as an additional dimension for the auditor style typology, since the empirical evidence that underpins the model does not give any information as to whether they are based on individual or group interviews. In the methodology literature, group interviews or focus groups have been distinguished from individual interviews in qualitative inquiries and evaluation [56–58]. The interactional construction of knowledge highlights the shift from an asymmetric power balance during the interview process, to the empowerment of the respondents. It is a shift from the operating principle of control, to collaboration between the interview participants [57], which could also be reflected upon in the ISO standards or guidelines**.** Concerns about interaction are important, because when health professionals participate in assessment contexts that are collaborative and supportive, it can self-reinforce a collaborative quality and safety culture [59]. Greenfield, Pawsey, and Braithwaite [59] also showed that auditors’ use of group interviews had positive effects for successful participation and collaboration with staff in accreditation. We suggest further qualitative explorative research to investigate into the role of the interactional element and how this influence both the certification processes and improvement efforts, as this is still an important area for knowledge generation. Methods wise we encourage in-depth studies of several certification bodies over time to identify variation in auditor work practice and style, and how their practices are considered from auditors and the certified bodies’ perspective. This may also help to further refine and extend the typology framework and judge whether all of the proposed styles actually exists in current auditing practices.

### Strength and limitations

The strengths of the study are the combination of observations and interviews behind the curtains of a certification body. The ability of follow auditors during certification processes and follow up with interviews provide a rich data set on their performance as observed and their perceived experiences. This is an approach lacking in the literature. This is an exploratory study, and the limitations of the study are first, a small sample size with three lead auditors in one certification body. However, choosing one certification body, allows for more details and adds to the knowledge about the practice of auditors representing one organization that may increase the information power [60] in the results. In addition, it is a challenge to involve additional certification bodies in a Norwegian context, due to the low number of certification bodies and thus a high transparency in this sector implying a risk of identifying participants. Second, a thematic analysis based on deductive approach could imply missing some of the inductive results. However, the framework we used in the deductive analysis contributed to guide the analysis and we were able to suggest further refinement of the typology which is often missing when using theory to guide research [61].

## Conclusions

Auditors from a certification body demonstrated a polymorphic certification reality in which guidance and stimulation for improvement were incorporated in the auditors’ styles and perceptions of ISO 9001 certification processes. This contrasts to what is often presumed as consistent, stringent and independent audit practices. A difficulty with the analytical auditor typology framework [24] is its lack of sensitivity to the different auditor styles. The originators of the typology suggest the need for more research to identify additional dimensions associated with the identified styles, like differences between the use of closed and open questions or examining the content and detail of note taking [24]. Our study suggests adding the distinctions between performing individual or group interviews during assessments, because these differences may influence the asymmetric power balances in the auditor-auditee encounter. However, there is a need for further research to understand and explore different auditor styles in different contextual settings and provide more knowledge on how auditor approaches affect the audit process and outcome in the organization under certification.

## Data Availability

The data generated and analyzed during this study are not publicly available due to concerns about confidentiality regarding a small sample size and the sensitive nature of the interviews. Instead, quotations and analytical categories are included in the text. We are happy to discuss the findings or the analysis if any questions arise.

## Abbreviation

ISO: International Organization for Standardization

## Footnotes

1 By the national recognized accreditation body Norsk Akkreditering, which verified the certification body’s independence and competence to carry out the certification process.
